# Biochemical Characteristics and PrP^Sc^ Distribution Pattern in the Brains of Cattle Experimentally Challenged with H-type and L-type Atypical BSE

**DOI:** 10.1371/journal.pone.0067599

**Published:** 2013-06-21

**Authors:** Grit Priemer, Anne Balkema-Buschmann, Bob Hills, Martin H. Groschup

**Affiliations:** 1 Institute of Novel and Emerging Infectious Diseases, Friedrich-Loeffler-Institut, Greifswald – Insel Riems, Germany; 2 Transmissible Spongiform Encephalopathy Secretariat, Health Canada, Ottawa, Ontario, Canada; USGS National Wildlife Health Center, United States of America

## Abstract

Besides the classical form of bovine spongiform encephalopathy (BSE) that has been known for almost three decades, two atypical forms designated H-type and L-type BSE have recently been described. While the main diagnostic feature of these forms is the altered biochemical profile of the accumulated PrP^Sc^, it was also observed in the initial analysis that L-type BSE displays a distribution pattern of the pathological prion protein (PrP^Sc^), which clearly differs from that observed in classical BSE (C-type). Most importantly, the obex region in the brainstem is not the region with the highest PrP^Sc^ concentrations, but PrP^Sc^ is spread more evenly throughout the entire brain. A similar distribution pattern has been revealed for H-type BSE by rapid test analysis. Based on these findings, we performed a more detailed Western blot study of the anatomical PrP^Sc^ distribution pattern and the biochemical characteristics (molecular mass, glycoprofile as well as PK sensitivity) in ten different anatomical locations of the brain from cattle experimentally challenged with H- or L-type BSE, as compared to cattle challenged with C-type BSE. Results of this study revealed distinct differences in the PrP^Sc^ deposition patterns between all three BSE forms, while the biochemical characteristics remained stable for each BSE type among all analysed brain areas.

## Introduction

Bovine spongiform encephalopathy (BSE) belongs to the so called “prion diseases”, also named transmissible spongiform encephalopathies (TSEs). These progressive neurodegenerative diseases are always fatal and can affect humans and animals. The most prominent TSE in humans is Creutzfeldt-Jakob disease (CJD). Scrapie in sheep and goats has been known for more than 250 years [1] while TSEs in other animals have only been described more recently. After the detection of the first BSE cases in the United Kingdom in 1986 [2], it was generally assumed for almost two decades that only one BSE strain existed in cattle. However in 2004, two different, so called atypical BSE strains were described in cattle in France [3] and Italy [4]. They were designated H- and L-type BSE, due to the molecular weight of the disease-associated prion-protein (PrP^Sc^) after protease degradation and Western blot analysis. The unglycosylated PrP fraction migrates 1–2 kDa higher in H-type BSE [3], and slightly lower in L-type BSE [4] as compared to classical (C-type) BSE. Until now, atypical BSE has been detected in more than 60 animals that were over eight years old in many countries across Europe, and also in Canada, the United States and Japan [5].

In earlier studies, we performed an initial analysis of the anatomical distribution of PrP^Sc^ depositions in the brains of experimentally challenged cattle using one of the approved BSE rapid test systems. The most prominent differences from the C-type BSE associated distribution pattern became obvious for the rhinencephalon, thalamus and basal nuclei, where distinct amounts of PrP^Sc^ could be detected in animals infected with both atypical BSE forms [6]. Moreover, it could be confirmed by different work groups that especially the obex region, representing the target area for all BSE diagnostic tests, showed relatively low amounts of PrP^Sc^ in cattle infected with L-type BSE [4], [7]–[9].

Comparative biochemical analyses of the disease-associated PrP^Sc^ in natural C-type, L-type and H-type BSE cases have revealed distinct differences in the PK cleavage sites and thus the antibody binding affinities, as well as in the proteinase K (PK) resistance at different pH conditions and PK concentrations [10], enabling a differentiation of the three BSE forms by Western blot analysis. Furthermore, the specific strain properties were confirmed after first and serial passages of atypical BSE in cattle by Western blot and immunohistochemistry (IHC) [11], [12].

Interestingly, for human TSEs, a variation of the glycoform ratio has been described in different brain regions for the sporadic form of Creutzfeldt-Jakob disease (sCJD) [13], [14], while for scrapie in sheep and mice, no such variability could be detected [15], [16] To our knowledge, no such study has ever been performed for BSE affected cattle. We therefore decided to also investigate the PrP^Sc^ ‘glycoprofiles’ in the brains of cattle experimentally challenged with atypical and classical BSE.

Moreover, transgenic mouse bioassays revealed that the biochemical BSE profile does not always remain stable, meaning that the specific PrP^Sc^ characteristics may change after passage in certain hosts. For example, a conversion of BASE (L-type BSE) to C-type BSE after serial transmission to inbred mice has been described [17]. Similar results were also reported in transgenic mice expressing ovine PrP [18]. Finally, others could demonstrate a comparable phenomenon for H-type BSE during serial passages in wild type mice with the emergence of classical BSE strain properties [19].

While earlier studies have mainly addressed the description of clinical signs [8], [9] and the overall biochemical and immunohistochemical strain properties [7]–[12] in cattle naturally and experimentally infected with H- and L-type BSE, this study has therefore focused on the determination of the quantity and quality of PrP^Sc^ depositions in ten different brain regions of four or five animals challenged with C-, H- or L-type BSE, as determined by Western blot analysis.

## Materials and Methods

### Experimental Animals

**Figure 1:**
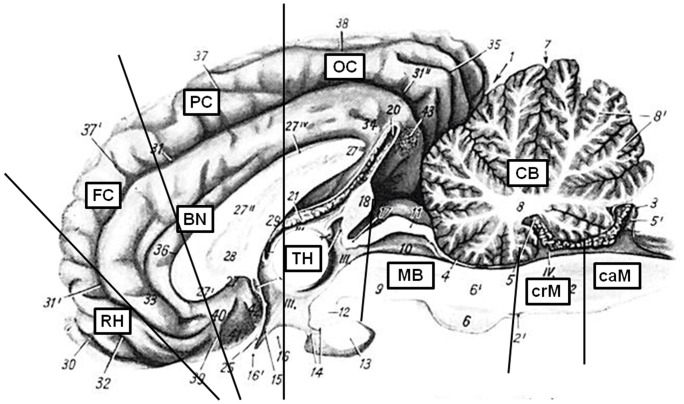
Analysed brain regions. This picture was modified after Nickel/Schummer/Seiferle [31]. Ten brain regions were analysed: CB – cerebellum, MB – midbrain including pons, crM – cranial medulla oblongata, caM – caudal medulla oblongata, RH – rhinencephalon (olfactory bulb), FC – frontal cortex, PC – parietal cortex, OC – occipital cortex, BN – basal nuclei, TH – thalamus. The medulla oblongata was separated into three regions, but due to the limited amount, only the cranial and caudal medulla could be investigated in detail.

Eleven female Holstein-Frisian calves (six month of age) were inoculated intracranially with 1 ml of a 10% (w/v) brain homogenate derived from the two identified German atypical BSE cases [20]. Six calves were challenged with L-type and five with H-type BSE. The details of this challenge study have been published elsewhere [8]. During necropsy, extensive sampling was carried out under TSE sterile conditions using a fresh single use instrument for every tissue sample collected, and also following a specific sampling order. Besides a variety of other samples from all organ systems, the following ten brain regions were collected: cerebellum, cranial medulla, caudal medulla, midbrain including pons, rhinencephalon, thalamus, basal nuclei, frontal cortex, parietal cortex and occipital cortex (Fig. 1). The brain was dissected sagitally and one half of the brain was formalin-fixed for histological examinations while the other half was frozen (-20°C and –70°C) for biochemical analysis. Only the brains of animals that had already developed moderate to severe BSE-specific clinical signs were included into this study, which is the reason why one of the cattle challenged with L-type BSE that was sacrificed five months after inoculation and was negative in all applied diagnostic tests, was not included in this detailed evaluation.

For a comparative analysis of brains from C-type affected cattle, we included the respective brain samples collected from four animals from an earlier oral challenge study with C-type BSE [21]. Two animals with moderate clinical signs (IT09, IT38) and two with severe clinical signs (IT22, IT49) were chosen, since the animals from the atypical BSE challenge study had also been in the moderate and severe clinical stages of the disease.

### Ethics statement

The challenge experiments in cattle described in this manuscript were approved by the competent authority of the Federal State of Mecklenburg-Western Pommerania, Germany on the basis of national and European legislation, namely the EU council directive 86/609/EEC for the protection of animals used for experiments (LALLF M-V/TSD/7221.3-020/06).

### Biochemical analysis of PrP^Sc^

First of all, the IDEXX HerdChek® enzyme immunoassay (EIA), which is one of the approved BSE rapid test systems, and which had been applied on four selected brain areas of L- and H-type affected cattle in an earlier study [6] was performed for all ten brain regions, according to the manufacturer’s instructions. Testing was done in duplicate. The optical density (OD) values determined for the cranial medulla of each BSE type was set at 100%, and the OD values determined for the other analysed brain regions were calculated in relation to this. The *cutoff* value was evaluated separately for every test run according to the manufacturer’s instructions (average mean of negative control + 0,120).

We also applied a highly sensitive PrP^Sc^ precipitation protocol using phosphotungstic acid with a subsequent Western blot (PTA-WB) using the PrP-specific monoclonal detection antibody mab L42 (r-biopharm, Darmstadt, Germany), as previously described [22]. This was carried out to determine the molecular weight, signal intensity and the so called `glycoprofilè (proportion of the di-, mono- and unglycosylated fraction) of the disease-associated PrP^Sc^.

The Western blot signals were analysed and quantified using the VersaDoc™ Imaging System (Bio-Rad, Munich, Germany). For the quantification of the signals, the signal intensity should be in the linear range of the VersaDoc™ Imaging System according to the manufacturer’s instructions. Therefore we first ran one initial gel for each sample to determine the signal intensity, followed by four gels with adjusted sample quantities that were used for our analysis. The molecular weight, the relative quantities and the volume (signal intensity) of each of the three PrP bands were determined using the Quantity One® Software. For the analysis of the glycoprofile, the signal intensity of each of the three PrP bands was calculated as the percentage of the overall signal for each brain region and BSE type individually.

For the quantitative analysis of the PrP^Sc^ depositions in the different brain regions, the cranial medulla was set as the reference (100%) and the signal intensities of the other brain regions were determined in relation to this. Arithmetic means were calculated for each sample.

Statistical analysis *(t-test)* was performed for the achieved WB data of the glycoprofile and the molecular mass.

### PK susceptibility

In a pilot experiment using homogenates from different brain regions, it was revealed that the PK susceptibility did not differ between the analysed brain areas. For the systematic analysis of the PK susceptibility, we therefore pooled different brain areas with the strongest signal intensities of each BSE form. The PK sensitivity was analysed by two different experimental setups: first, we added varying PK concentrations of 0, 25, 50, 250 or 1000 µg/ml to the reaction and incubated these for 60 min at 55°C. The remaining PrP^Sc^ signal determined after incubation with a final PK concentration of 50 µg/ml, which is used in our standard protocol, was set as the reference point (100%). In the second approach, we incubated reactions with 50 µg/ml PK for 1, 3, 6, 10 and 26 hours. Here, the residual signal obtained after a one hour PK digestion using 50 µg/ml (standard protocol) was again set as the reference point (100%).

For each BSE type, testing was done in duplicate, the gel electrophoresis was repeated three times and the arithmetic means of the obtained signal intensities were calculated.

## Results

This study was aimed at the detailed analysis of the anatomical distribution and the biochemical characteristics of both atypical BSE forms designated L-type and H-type, as compared to C-type BSE.

**Figure 2:**
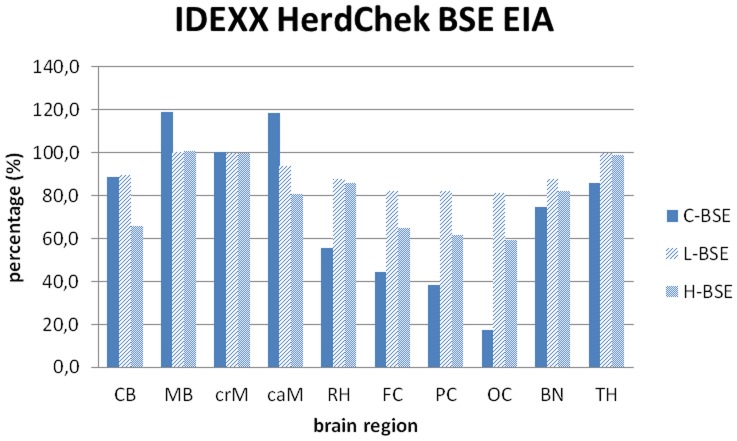
PrP^Sc^ distribution pattern determined by IDEXX HerdChek® EIA. Results as determined for the different brain areas using the approved BSE rapid test, performed according to the manufacturer’s instructions. Testing was done in duplicate and the arithmetic means were calculated for the individual BSE types. Cranial medulla was set as the reference (100%) and the other brain regions were set in relation to it. CB – cerebellum, MB – midbrain including pons, crM – cranial medulla oblongata, caM – caudal medulla oblongata, RH – rhinencephalon (olfactory bulb), FC – frontal cortex, PC – parietal cortex, OC – occipital cortex, BN – basal nuclei, TH – thalamus.

At first we used the IDEXX HerdChek® EIA rapid test on the ten brain areas analysed in this study. In cattle challenged with L-type BSE (*n* = 5) the highest OD values were detected in the midbrain, cranial medulla and thalamus. The OD values determined for these examined brain regions showed relatively even overall OD values ranging between 2.47 and 3.05, which is close to the upper limit of the test, and therefore in the non-linear range. In cattle challenged with H-type BSE (*n* = 5) the highest OD values could also be detected in the midbrain, cranial medulla and thalamus. Like in L-type BSE affected cattle, the OD values were relatively even, but overall lower than determined for L-type BSE, ranging between 1.60 and 2.68. In contrast to the atypical cases, a more distinct difference was seen between the different brain areas of cattle affected with C-type BSE. For the medulla and midbrain region OD values between 2.61 and 3.09 were determined, as opposed to the OD values for the cortex regions between 0.46 and 1.16. The relative signals in relation to the OD value determined for the cranial medulla as determined by the IDEXX HerdChek® EIA are summarized in Fig. 2.

**Figure 3:**
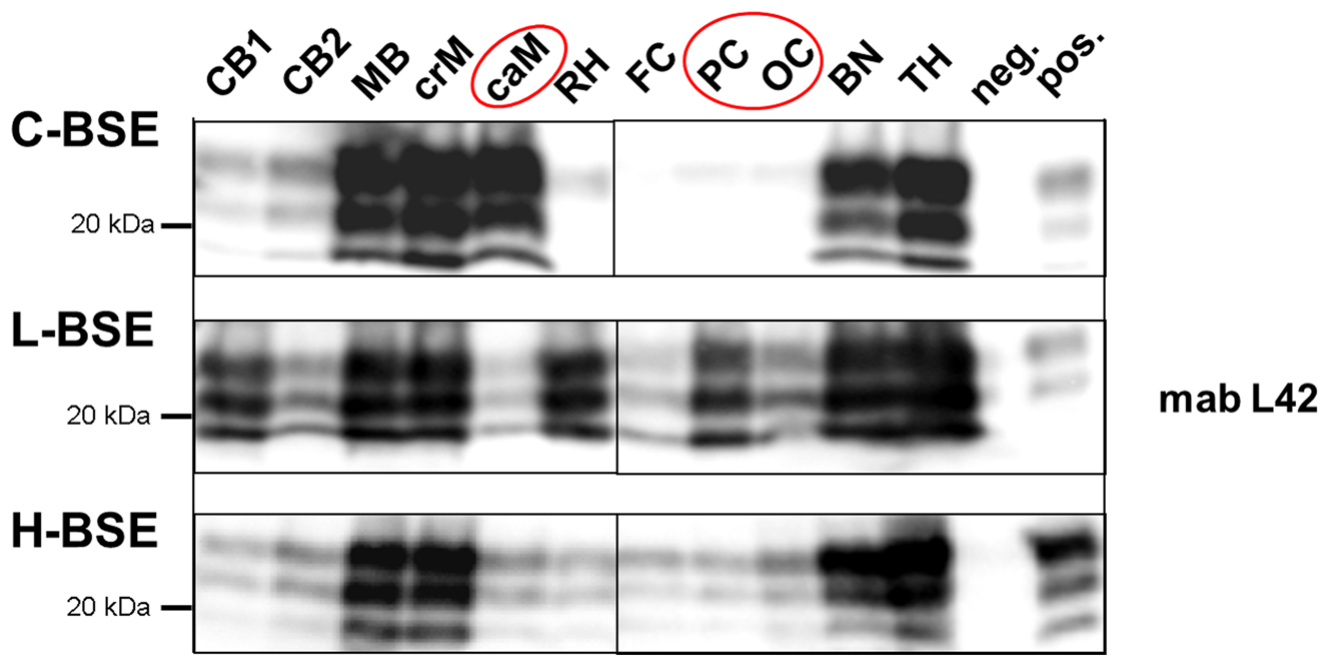
PrP^Sc^ distribution pattern determined by PTA-WB. Western blots analysis using mab L42 as the detection antibody of ten brain regions of cattle clinically affected with C-, L- or H-type BSE. The figure displays three representative experimental animals (one of each BSE form), all in the final clinical stage of the disease (++ moderate to +++ severe clinical signs). CB – cerebellum, MB – midbrain including pons, crM – cranial medulla oblongata, caM – caudal medulla oblongata, RH – rhinencephalon (olfactory bulb), FC – frontal cortex, PC – parietal cortex, OC – occipital cortex, BN – basal nuclei, TH – thalamus.

**Figure 4:**
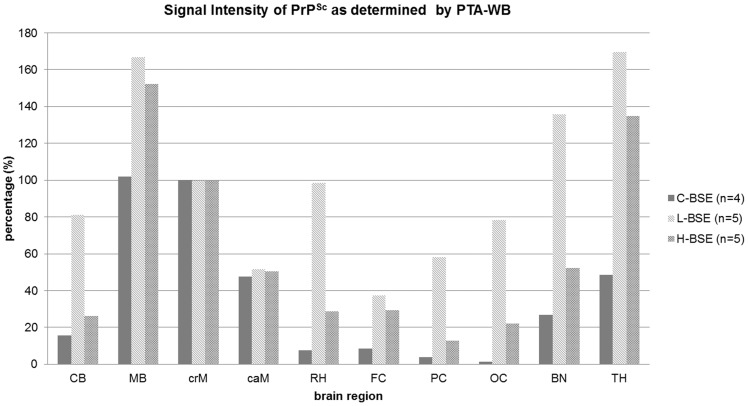
Signal Intensity of PTA-WB signals. Western blot signals were measured with a digital camera (VersaDoc™) and evaluated with the respective software (Quantity One®, BioRad, Munich, Germany). The arithmetic means were calculated for the individual BSE types. Cranial medulla was set as the reference (100%) and the other brain regions were set in relation to it. CB – cerebellum, MB – midbrain including pons, crM – cranial medulla oblongata, caM – caudal medulla oblongata, RH – rhinencephalon (olfactory bulb), FC – frontal cortex, PC – parietal cortex, OC – occipital cortex, BN – basal nuclei, TH – thalamus.

Comparable distribution patterns were determined when analysing the same samples by PTA-WB (Fig. 3). However, this method allowed for a more exact quantification of the signal intensities, since the Western blot signals were adjusted to be in the linear range of the VersaDoc™ Imaging System beforehand. In cattle affected with H- and L-type atypical BSE, PrP^Sc^ was found to be spread more evenly throughout the whole brain than in C-type BSE affected cattle. The deposition patterns were similar in L- and H-type BSE, but L-type BSE displayed distinctly stronger overall intensities. Analysis of the Western blot signal intensities as compared to the intensity determined for the cranial medulla (set at 100%) revealed high values for L-type BSE in every brain region, which were distinctly higher compared to the other two BSE forms (Fig. 4). Thereby, the highest concentrations of PrP^Sc^ could be detected in the brainstem, basal nuclei, thalamus and rhinencephalon, while H-type BSE infected cattle displayed the highest PrP^Sc^ concentrations in the brainstem and thalamus. Classical BSE affected animals exhibited lower signal intensities in all brain regions as compared to atypical BSE, with the highest concentrations located in the brainstem. In summary, we were able to identify the three analysed cortex regions as the areas displaying the most obvious differences when comparing classical to atypical BSE. In contrast to classical BSE, both atypical BSE forms are characterized by considerable amounts of PrP^Sc^ in the cortex regions.

**Figure 5:**
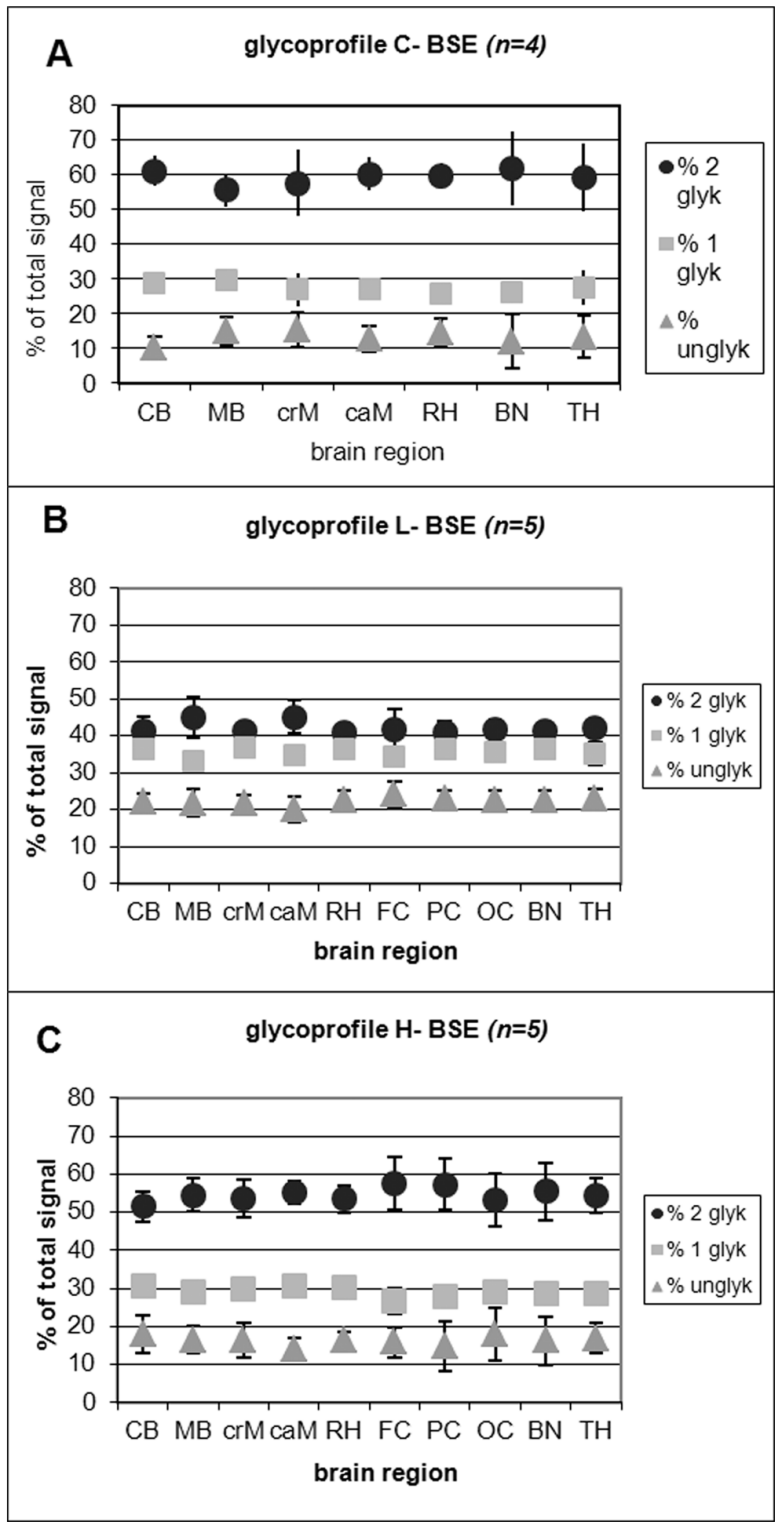
‘Glycoprofile’ analysis of C-, L- and H-type BSE. The relative quantity of the three PrP^Sc^ bands was determined using the Quantity One® Software after background subtraction for the glycosylation profile analysis (percentage of the di-, mono- and unglycosylated PrP^Sc^ band). All values were calculated as means ± standard deviations of four SDS-PAGE runs. Since it was not possible for classical BSE to detect the TSE-associated PrP^Sc^ triplet banding pattern after PK digestion in the three cortex regions, these regions could not be evaluated. (A) the glycoprofile of the classical BSE (C-type BSE), (B) the glycoprofile of the L-type BSE and (C) the glycoprofile of the H-type BSE. CB – cerebellum, MB – midbrain including pons, crM – cranial medulla oblongata, caM – caudal medulla oblongata, RH – rhinencephalon (olfactory bulb), FC – frontal cortex, PC – parietal cortex, OC – occipital cortex, BN – basal nuclei, TH – thalamus.

Analysis of the glycosylation profile (**“**glycoprofile**”**) determined the mean percentage ratio of the di-, mono- and unglycosylated PrP^Sc^ fraction out of the overall signal. Our study revealed glycoprofiles for classical BSE of 60 : 27 : 13%, for L-type BSE 42 : 36 : 22% and for H-type BSE 55 : 29 : 16% for the diglycosylated : monoglycosylated : unglycosylated PrP^Sc^ fraction (Fig. 5).

The mean value of the molecular masses of the unglycosylated isoform of PrP^Sc^ of all investigated samples was determined for C-type as 17.61±0.25 kDa, for L-type as 17.59±0.10 kDa and for H-type BSE as 18.69±0.16 kDa.

While we determined distinct differences in the glycoprofiles and the molecular masses of the PrP^Sc^ fractions analysed for C-type, L-type and H-type BSE, we however did not observe any obvious variations in these characteristics among the analysed brain areas of each BSE type. This finding was confirmed by statistical analysis *(t-test).*

Moreover, all cattle of a group challenged with one BSE type revealed the same PrP^Sc^ deposition pattern of the inoculum in our analysis; hence no alteration of the specific biochemical characteristics had occurred for the three BSE strains upon subpassage in cattle.

**Figure 6:**
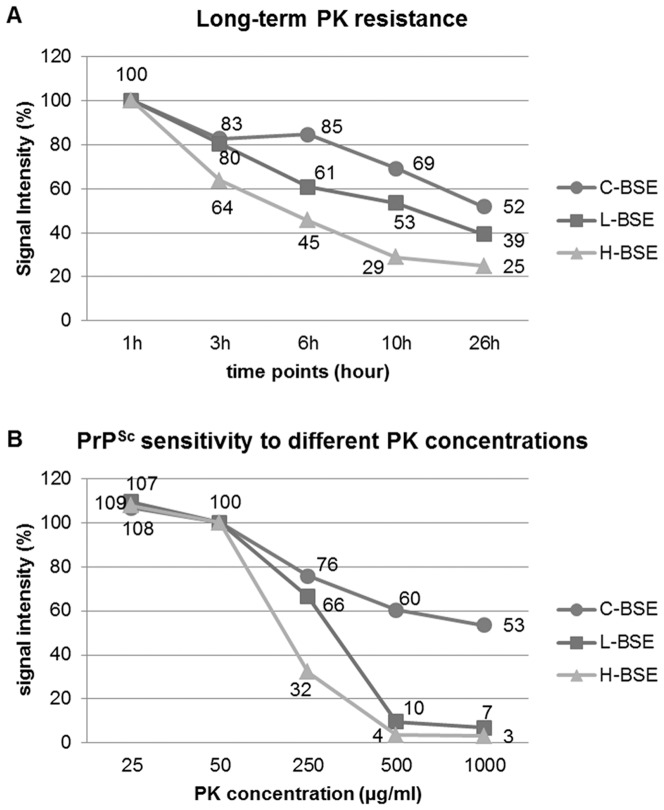
Susceptibility to PK digestion. PK susceptibility was analyzed by PTA-WB. (A) Influence of final PK concentrations: 0, 25, 50, 250, 500 and 1000 µg/ml. The intensity of the Western blot signals were determined, whereby 50 µg/ml, which is the standard PK concentration used in our protocol, was set as a reference point (100%). PK digestion was performed for 60 min at 55°C. (B) Degradation of the PrP^Sc^ in accordance to the length of PK digestion between 1 and 26 hours. 50 µg/ml PK was applied in all of the experiments. Signal intensity was measured and the signal obtained after 60 min PK digestion (standard protocol) was set as the reference point (100%).

With the evaluation of the PK susceptibility of the three BSE forms by applying different PK concentrations (Fig. 6A) and long-term PK incubations (Fig. 6B) we were able to define distinct patterns of proteinase K degradation for all three BSE forms. All three showed only minimal variations of the residual signal intensity with PK concentrations between 25 µg/ml and 50 µg/ml. Distinct differences were seen when PK concentrations of 250 µg/ml or higher were applied. With a five times higher PK concentration than normally used in our protocol (i.e. 250 µg/ml), we observed a reduction of the signal intensity for C-type of up to 25%, for L-type of up to 35%, and more notably H-type BSE was reduced by over 66%. With a PK concentration 20 times higher than what is normally used (i.e. 1000 µg/ml), we found a reduction in the signal intensity of approximately 50% for C-Type BSE, whereas the signals for both atypical BSE types were reduced by 93–97%, showing a residual signal intensity of only 3–7% compared to that of the signals obtained after incubation with 50 µg/ml PK. In conclusion, we could illustrate for classical BSE only a slow degradation of the pathological prion protein by PK digestion for all five investigated PK concentrations, which never resulted in a reduction of the signal intensity by more than 50%, even with a final PK concentration of 1000 µg/ml. In contrast, the pathological prion protein associated to both atypical BSE strains was almost completely degraded under such stringent PK concentrations.

In addition, when we applied a long-term PK digestion (Fig. 6B), the differences between the two atypical BSE strains became even more obvious. After a three hour digestion with PK, the signal for C- and L-type BSE were reduced by approximately 20%, whereas H-type BSE was already reduced by up to 35%. After a six hour incubation, the C-type BSE signal was still only reduced by 20% while the signal for L-type BSE was now reduced by 40% and H-type was even further reduced by 55%. Finally, after an incubation of 26 hours, the signal intensity of C-type BSE was now reduced by almost 50%, L-type BSE by 60%, and H-type BSE was reduced by more than 70%.

## Discussion

In the present study, we assessed the anatomical distribution pattern and the biochemical characteristics of the accumulated PrP^Sc^ in ten different brain regions of cattle affected with atypical H- and L-type BSE, as compared to that of C-type BSE. All animals were in the moderate to severe clinical stage of a BSE infection when euthanized. For a comparison of the results obtained after an intracerebral challenge with atypical BSE to those of classical BSE, we selected four representative animals from the oral C-type BSE pathogenesis study performed earlier [21]. For the L- and H-type BSE challenge, the intracerebral transmission route had been chosen, because only low quantities from the brains of the two atypical BSE field cases identified in Germany were available [20]. In a recent publication by Fukuda and his colleagues, the anatomical distribution of PrP^Sc^ depositions after intracranial challenged with C-type BSE was assessed in 18 different brain regions by Western blot [23]. The results presented for nine cattle that had been sacrificed in the clinical stage of the disease BSE show a very good correlation with what has been determined in this study (Table 1 *)*. However, it reveals that the cortex areas, especially the occipital cortex, seem to be less affected in orally challenged cattle. On the other hand, the Italian L-type field cases where different brain areas were available for analysis also displayed high PrP^Sc^ concentrations in the cortex [4], suggesting that this is a feature of L-type BSE. Still, the results determined for the cortex samples in our study should be interpreted cautiously. Moreover, for BSE challenged sheep, a detailed study of the injection site after intracerebral inoculation dismissed the initial hypothesis from the same authors postulated in an earlier publication [24], that after intracerebral inoculation, the quantity of PrP^Sc^ depositions was higher around the injection canal. It could even be further demonstrated by the use of bovine- versus ovine-PrP-specific antibodies, that the PrP^Sc^ depositions around the injection site were indeed newly formed ovine PrP^Sc^ formations, rather than the bovine inoculum [25]. The only effect that may be attributed to the intracerebral inoculation is a somewhat higher quantity of PrP^Sc^ depositions in the forebrain areas [25], which would be represented by the rhinencephalon sample analysed in our study. In the same study, it was established that following an intracranial inoculation of sheep, PrP^Sc^ and infectivity are usually washed out from the damaged brain area via the blood stream and dispersed into the periphery [25]. Subsequently propagated infectivity is then slowly ascending again into the CNS probably via neuronal routes [26]. We therefore conclude that the results determined for cattle challenged intracerebrally with atypical BSE can generally be put into relation to data generated from cattle challenged orally with C-type BSE.

**Table 1:** Comparison of PrP^Sc^ distribution patterns in C-type BSE affected cattle after oral versus intracerebral challenge.

Case No.	IT09	IT22	IT38	IT49	8	12	15	16
	oral	oral	oral	oral	i.c. *	i.c.*	i.c.*	i.c.*
*mpi*	*32*	*44*	*44*	*36*	*20*	*23*	*24*	*24*
CB	+	+	+	+	+	++	++	++
MB	++	++	++	++	++	++	++	++
crM	+	++	++	++	++**	++**	++**	++**
caM	++	+	++	++	n.a.	n.a.	n.a.	n.a.
RH	–	+	+	+	n.a.	n.a.	n.a.	n.a.
FC	+	+	–	–	++	++	–	+
PC	–	–	–	–	+	++	+	+
OC	–	–	–	–	++	++	++	+
BN	+	+	–	++	++***	++***	–***	++***
TH	–	+	+	++	++	++	–	++

Data from orally challenged animals IT09, IT22, IT38, IT49 [21] were assembled with data from intracerebrally challenged animals termed case no. 8, 12, 15, 16 [23]. Signal intensities described in [23] and determined in this study were quantified as follows: – no PrP^Sc^ detectable, +: moderate PrP^Sc^ depositions, ++ strong PrP^Sc^ depositions.

i.c.  =  intracerebral inoculation.

* Fukuda et al., 2012 [23].

** Obex.

*** Accumbens.

We performed biochemical analyses of the PrP^Sc^ depositions in ten different brain regions. These analyses verified distinct qualitative and quantitative differences in the anatomical distribution of PrP^Sc^ depositions in the brains of cattle clinically affected with C-, L- or H-type BSE. While for C-type BSE the PrP^Sc^ depositions are mainly restricted to the brainstem and to a lesser extent to the cerebellum, whereas cattle affected with both atypical BSE forms showed an almost even distribution of the pathological prion protein throughout the whole brain. Although deposition patterns were similar for L- and H-type BSE, L-type BSE displayed stronger overall intensities. Even though the results from these areas may be biased by the inoculation route as explained above, the most pronounced differences were detected in the cortex regions. While cattle affected with C-type BSE showed only faint signals, even in the end-stage of the disease, cattle affected with both atypical BSE forms displayed distinctly higher PrP^Sc^ concentrations in the analysed cortex samples. Cattle affected with all three BSE types showed remarkably high PrP^Sc^ amounts in the midbrain, medulla oblongata and in the thalamus. However, higher concentrations were determined in the caudal medulla than in cranial medulla for classical BSE. The opposite was true for atypical BSE, where higher PrP^Sc^ quantities were found in the cranial medulla as opposed to the caudal medulla. This finding stresses the importance to carefully take the diagnostic sample from the obex, instead of caudal medulla for BSE rapid testing, in particular with regard to L-type BSE, because the thalamus, basal nuclei and midbrain showed stronger involvement than the obex and especially the caudal medulla. This confirms similar findings that have been reported by others [7].

The IDEXX HerdChek® EIA is one of the EU-approved rapid tests with a comparably high sensitivity for all three BSE forms [27]. Insofar, we noticed that the optical density values for L-type BSE were all close to the saturation limit and therefore in the non-linear range of the test. A serial dilution approach could help to elucidate the quantity of PrP^Sc^ depositions also in L-type BSE affected cattle. However, in our approach we did not use diluted samples in the BSE rapid test, but we adjusted the protein amounts in the Western blot, so that we were able to quantify the PrP^Sc^ amounts in this assay.

During the challenge experiment with atypical BSE in cattle, we observed behavioural differences between classical and atypical BSE affected cattle during the end-stage of disease which have already been described in more detail [9]. We noticed that cattle clinically affected with atypical BSE became lethargic and depressive and isolated themselves from the rest of the herd. However, when manipulated during the clinical analysis, these animals turned out to suffer from hind limb ataxia, hyperesthesia (especially in the face) and anxiety, very similar to animals affected with classical BSE. These observations are supported by other reports, where L-type BSE affected cattle showed dullness, depression, exaggerated response to facial touch [28], an initial over-reactivity to tactile stimuli which later yielded a more dull form [9], and a distinct loss of body condition [11]. We therefore postulate that the classical BSE symptoms may be masked or overruled by the depression and loss of condition that at first sight is typical for H- and L-type BSE. This depression may well be caused by the higher grade of involvement of the cerebral cortex in both atypical BSE forms. The different clinical picture may therefore be anchored in the function and phylogenesis of the different involved brain regions.

We then analysed the glycosylation profiles and molecular masses of PK digested PrP^Sc^ accumulated in the ten different brain regions. As expected non-glycosylated PrP^Sc^ of L-type BSE had a slightly lower molecular mass than that of C-type BSE, whereas non-glycosylated PrP^Sc^ of H-type was approximately one kDa higher and all three BSE forms were encoded by a distinct glycoform. However, apart from these general differences, no statistically significant sampling effects were revealed for any of the three BSE types. These findings confirm what has been reported for mouse passaged BSE and scrapie strains [15] and for scrapie in sheep [16], while regional differences have been reported for sCJD [13], [14].

Furthermore, we could show that PrP^Sc^ associated with C-type BSE is more resistant towards PK degradation than that of both atypical BSE forms, irrespective of the experimental setup addressing a PK digestion with higher concentrations of up to 1000 µg/ml, or a long-term PK digestion of up to 26 hours. These findings confirm earlier reports [9], [10] regarding the proteinase K (PK) susceptibility of the three BSE forms. These results might have an impact on the actual persistence of BSE infectivity in the environment, since ubiquitous proteases will be more efficient in the degradation of atypical BSE-derived PrP^Sc^ as opposed to C-type BSE. Although it has been postulated that PrP^Sc^ is not identical to the infectious agent [29], [30], a reduction of the accumulated PrP^Sc^ will also reduce the infectivity titre in a sample.

## Conclusions

In summary, we could show distinct differences in the anatomical distribution of PrP^Sc^ deposition in the brains of cattle affected with the three BSE forms. These differences are sufficiently clear to enable discrimination between classical BSE and atypical BSE, and at least supportive for a differentiation of both atypical BSE forms by Western blot or ELISA rapid test analysis, providing a sufficient number of relevant brain areas are available for analysis. Moreover, these findings are scientifically interesting, since we believe that they mirror the clinical picture observed in cattle affected with either classical or atypical BSE. The differences in protease sensitivity may also have a practical impact on the stability in the environment and possibly on the interspecies transmissibility of the three BSE forms, but these hypothesis need to be elucidated by further analysis.

## References

[1] McGowan J (1922) Scrapie in sheep. Scott J Agric 5: 365–375.

[2] Wells GA, Scott AC, Johnson CT, Gunning RF, Hancock RD, et al (1987) A novel progressive spongiform encephalopathy in cattle. Vet Rec 121: 419–420. 3424605 10.1136/vr.121.18.419

[3] Biacabe AG, Laplanche JL, Ryder S, Baron T (2004) Distinct molecular phenotypes in bovine prion diseases. EMBO Rep 5: 110–115. 14710195 10.1038/sj.embor.7400054 PMC1298965

[4] Casalone C, Zanusso G, Acutis P, Ferrari S, Capucci L, et al (2004) Identification of a second bovine amyloidotic spongiform encephalopathy: molecular similarities with sporadic Creutzfeldt-Jakob disease. Proc Natl Acad Sci U S A 101: 3065–3070. 14970340 10.1073/pnas.0305777101 PMC365745

[5] Anonym (2012) Report on the monitoring of ruminants for the presence of Transmissible Spongiform Encephalopathies (TSEs) in the EU in 2011. © European Union, 2012.

[6] Balkema-Buschmann A, Fast C, Kaatz M, Eiden M, Ziegler U, et al (2011) Pathogenesis of classical and atypical BSE in cattle. Prev Vet Med 102: 112–117. 21592603 10.1016/j.prevetmed.2011.04.006

[7] Polak MP, Zmudzinski JF (2012) Distribution of a pathological form of prion protein in the brainstem and cerebellum in classical and atypical cases of bovine spongiform encephalopathy. Vet J 191: 128–130. 21277240 10.1016/j.tvjl.2010.12.019

[8] Balkema-Buschmann A, Ziegler U, McIntyre L, Keller M, Hoffmann C, et al (2011) Experimental challenge of cattle with German atypical bovine spongiform encephalopathy (BSE) isolates. J Toxicol Environ Health A 74: 103–109. 21218339 10.1080/15287394.2011.529060

[9] Konold T, Bone GE, Clifford D, Chaplin MJ, Cawthraw S, et al (2012) Experimental H-type and L-type bovine spongiform encephalopathy in cattle: observation of two clinical syndromes and diagnostic challenges. BMC Vet Res 8: 22. 22401036 10.1186/1746-6148-8-22 PMC3378435

[10] Jacobs JG, Langeveld JP, Biacabe AG, Acutis PL, Polak MP, et al (2007) Molecular discrimination of atypical bovine spongiform encephalopathy strains from a geographical region spanning a wide area in Europe. J Clin Microbiol 45: 1821–1829. 17442800 10.1128/JCM.00160-07 PMC1933055

[11] Okada H, Iwamaru Y, Imamura M, Masujin K, Matsuura Y, et al (2011) Experimental H-type bovine spongiform encephalopathy characterized by plaques and glial- and stellate-type prion protein deposits. Vet Res 42: 79. 21699704 10.1186/1297-9716-42-79 PMC3132711

[12] Okada H, Iwamaru Y, Kakizaki M, Masujin K, Imamura M, et al (2012) Properties of L-type bovine spongiform encephalopathy in intraspecies passages. Vet Pathol 49: 819–823. 22081134 10.1177/0300985811427150

[13] Levavasseur E, Laffont-Proust I, Morain E, Faucheux BA, Privat N, et al (2008) Regulating factors of PrP glycosylation in Creutzfeldt-Jakob disease--implications for the dissemination and the diagnosis of human prion strains. PLoS One 3: e2786. 18665216 10.1371/journal.pone.0002786 PMC2464735

[14] Parchi P, Strammiello R, Notari S, Giese A, Langeveld JP, et al (2009) Incidence and spectrum of sporadic Creutzfeldt-Jakob disease variants with mixed phenotype and co-occurrence of PrPSc types: an updated classification. Acta Neuropathol 118: 659–671. 19718500 10.1007/s00401-009-0585-1 PMC2773124

[15] Kuczius T, Haist I, Groschup MH (1998) Molecular analysis of bovine spongiform encephalopathy and scrapie strain variation. J Infect Dis 178: 693–699. 9728537 10.1086/515337

[16] Sweeney T, Kuczius T, McElroy M, Gomez Parada M, Groschup MH (2000) Molecular analysis of Irish sheep scrapie cases. J Gen Virol 81: 1621–1627. 10811947 10.1099/0022-1317-81-6-1621

[17] Capobianco R, Casalone C, Suardi S, Mangieri M, Miccolo C, et al (2007) Conversion of the BASE prion strain into the BSE strain: the origin of BSE? PLoS Pathog 3: e31. 17352534 10.1371/journal.ppat.0030031 PMC1817656

[18] Beringue V, Andreoletti O, Le Dur A, Essalmani R, Vilotte JL, et al (2007) A bovine prion acquires an epidemic bovine spongiform encephalopathy strain-like phenotype on interspecies transmission. J Neurosci 27: 6965–6971. 17596445 10.1523/JNEUROSCI.0693-07.2007 PMC6672218

[19] Baron T, Vulin J, Biacabe AG, Lakhdar L, Verchere J, et al (2011) Emergence of classical BSE strain properties during serial passages of H-BSE in wild-type mice. PLoS One 6: e15839. 21264286 10.1371/journal.pone.0015839 PMC3021503

[20] Buschmann A, Gretzschel A, Biacabe AG, Schiebel K, Corona C, et al (2006) Atypical BSE in Germany--proof of transmissibility and biochemical characterization. Vet Microbiol 117: 103–116. 16916588 10.1016/j.vetmic.2006.06.016

[21] Hoffmann C, Eiden M, Kaatz M, Keller M, Ziegler U, et al (2011) BSE infectivity in jejunum, ileum and ileocaecal junction of incubating cattle. Vet Res 42: 21. 21314904 10.1186/1297-9716-42-21 PMC3048543

[22] Kaatz M, Fast C, Ziegler U, Balkema-Buschmann A, Hammerschmidt B, et al (2012) Spread of classic BSE prions from the gut via the peripheral nervous system to the brain. Am J Pathol 181: 515–524. 22781833 10.1016/j.ajpath.2012.05.001

[23] Fukuda S, Onoe S, Nikaido S, Fujii K, Kageyama S, et al (2012) Neuroanatomical distribution of disease-associated prion protein in experimental bovine spongiform encephalopathy in cattle after intracerebral inoculation. Jpn J Infect Dis 65: 37–44. 22274156

[24] Gonzalez L, Martin S, Houston FE, Hunter N, Reid HW, et al (2005) Phenotype of disease-associated PrP accumulation in the brain of bovine spongiform encephalopathy experimentally infected sheep. J Gen Virol 86: 827–838. 15722546 10.1099/vir.0.80299-0

[25] Siso S, Jeffrey M, Martin S, Houston F, Hunter N, et al (2009) Pathogenetical significance of porencephalic lesions associated with intracerebral inoculation of sheep with the bovine spongiform encephalopathy (BSE) agent. Neuropathol Appl Neurobiol 35: 247–258. 19207266 10.1111/j.1365-2990.2009.01013.x

[26] Wells GA, Spiropoulos J, Hawkins SA, Ryder SJ (2005) Pathogenesis of experimental bovine spongiform encephalopathy: preclinical infectivity in tonsil and observations on the distribution of lingual tonsil in slaughtered cattle. Vet Rec 156: 401–407. 15816193 10.1136/vr.156.13.401

[27] Meloni D, Davidse A, Langeveld JP, Varello K, Casalone C, et al (2012) EU-approved rapid tests for bovine spongiform encephalopathy detect atypical forms: a study for their sensitivities. PLoS One 7: e43133. 22984410 10.1371/journal.pone.0043133 PMC3439472

[28] Lombardi G, Casalone C, D'Angelo A, Gelmetti D, Torcoli G, et al (2008) Intraspecies transmission of BASE induces clinical dullness and amyotrophic changes. PLoS Pathog 4: e1000075. 18497860 10.1371/journal.ppat.1000075 PMC2374911

[29] Lasmezas CI, Deslys JP, Robain O, Jaegly A, Beringue V, et al (1997) Transmission of the BSE agent to mice in the absence of detectable abnormal prion protein. Science 275: 402–405. 8994041 10.1126/science.275.5298.402

[30] Balkema-Buschmann A, Eiden M, Hoffmann C, Kaatz M, Ziegler U, et al (2011) BSE infectivity in the absence of detectable PrP(Sc) accumulation in the tongue and nasal mucosa of terminally diseased cattle. J Gen Virol 92: 467–476. 20943888 10.1099/vir.0.025387-0

[31] Nickel R, Schummer A, Seiferle E (1992) Lehrbuch der Anatomie der Haustiere. PAREY Band IV: S.68.

